# Recent Insight into the Genetic Basis, Clinical Features, and Diagnostic Methods for Neuronal Ceroid Lipofuscinosis

**DOI:** 10.3390/ijms23105729

**Published:** 2022-05-20

**Authors:** Konrad Kaminiów, Sylwia Kozak, Justyna Paprocka

**Affiliations:** 1 Students’ Scientific Society, Department of Pediatric Neurology, Faculty of Medical Sciences in Katowice, Medical University of Silesia, 40-752 Katowice, Poland; kaminiow.k@gmail.com (K.K.); sylwiakozak@icloud.com (S.K.); 2 Pediatric Neurology Department, Faculty of Medical Sciences in Katowice, Medical University of Silesia, 40-752 Katowice, Poland

**Keywords:** neuronal ceroid lipofuscinosis, NCL, neurodegenerative disorders, lysosomal storage disorders, genetics

## Abstract

Neuronal ceroid lipofuscinoses (NCLs) are a group of rare, inherited, neurodegenerative lysosomal storage disorders that affect children and adults. They are traditionally grouped together, based on shared clinical symptoms and pathological ground. To date, 13 autosomal recessive gene variants, as well as one autosomal dominant gene variant, of NCL have been described. These genes encode a variety of proteins, whose functions have not been fully defined; most are lysosomal enzymes, transmembrane proteins of the lysosome, or other organelles. Common symptoms of NCLs include the progressive loss of vision, mental and motor deterioration, epileptic seizures, premature death, and, in rare adult-onset cases, dementia. Depending on the mutation, these symptoms can vary, with respect to the severity and onset of symptoms by age. Currently, all forms of NCL are fatal, and no curative treatments are available. Herein, we provide an overview to summarize the current knowledge regarding the pathophysiology, genetics, and clinical manifestation of these conditions, as well as the approach to diagnosis.

## 1. Introduction

The term neuronal ceroid lipofuscinoses (NCL) refers to a group of autosomal recessive neurodegenerative disorders, presenting with myoclonic epilepsy, psychomotor delay, progressive loss of vision, and early death [1,2,3,4]. It is worth noting that NCL are the most common cause of childhood dementia [3,5,6]. Cardinal to NCL pathology is the toxic levels of protein aggregates in the central nervous system (CNS) [4,5,7], which, more specifically, are aggregates of lipopigments (lipofuscin) within lysosomes [4,8,9]. They are the most prevalent neurodegenerative disorders of childhood, with an incidence in the USA estimated at 1.6–2.4/100,000 [2,4,10,11,12], while, in Scandinavian countries, the incidence varies between: 2–2.5/100,000 in Denmark [2,10,11], 2.2/100,000 in Sweden [2,10,11], 3.9/100,000 in Norway [2,10,11], 4.8/100,000 in Finland [2,10,12], and 7/100,000 in Iceland [2,10]. The rarity of incidence and scarcity of the disease models have limited comprehensive understanding of the pathological factors that lead to disease progression. To date, 13 autosomal recessive, as well as one autosomal dominant gene, variants of NCL have been described, each involving functional defects in lysosomal protein [3,4,13,14,15,16]. Aggregated lysosomal lipofuscin affects the neuronal cytoskeleton and cellular trafficking, resulting in neuronal loss and pathological glial proliferation and activation [4,7]. More specifically, defective proteins (corresponding genes designated *CLN* for “ceroid lipofuscinosis, neuronal”) include secreted lysosomal proteins (CLN1/PPT1, CLN2/TPP1, CLN5, CLN10/CTSD, CLN13/CTSF, and CLN11/GRN) or membrane proteins (CLN3, CLN7/MFSD8, and CLN12/ATP13A2) [4,17,18,19,20]. Two of them are the membrane proteins of the endoplasmic reticulum (CLN6 and CLN8). Other NCL proteins are cytoplasmic (CLN4/DNAJC5 and CLN14/KCTD7), which peripherally associate with cell membranes [4,17,18,19,20].

NCLs are grouped on pathological grounds, due to the common presence of the neuronal and extra-neuronal accumulations of autofluorescent pigments, despite the different underlying biochemical etiologies [2,3,21]. In all forms of NCL, lipopigment storage material accumulates in macrophages, neurons, and some somatic tissues, including vascular endothelial cells and smooth muscle cells [3,22]. Neuronal loss is profound and extensive in most patients with NCL, leading to cortical gray matter atrophy, cerebellar atrophy, and secondary ventricular enlargement, all of which progress throughout the course of the disease [3,22,23]. The degree of atrophy and ventricular enlargement varies with the form of NCL [3,22,23]. The prominent activation of microglia and astrocytes precedes (and perhaps causes) neuronal loss, accurately predicting its distribution [3,24,25,26].

NCL is classified into five primary types, as shown in Table 1.

The most common NCL types are the CLN1 (also known as “Infantile NCL”), CLN2 (“Late Infantile”), CLN3 (“Juvenile NCL”), and CLN6 (“variant LINCL”) diseases [4,17,18,19]. Table 2 shows all known types of neuronal ceroid lipofuscinoses, together with the genes whose mutations are responsible for their occurrence. Table 2 also contains the age of onset of the disease and the proteins whose functions are impaired.

In 1998, a database was established that includes published mutations and sequence changes in genes causing NCL, together with unpublished data, included with permission. All mutation details can be found in the publicly available NCL mutation database at the following Internet address: www.ucl.ac.uk/ncl-disease (accessed on 16 May 2022).

## 2. Genetics and Pathophysiology

### 2.1. Animal Models in NCL Pathology

The development of various animal models has provided scientists with a range of tools for studying the effects of the mutations responsible for NCL. In NCL, as in many other neurodegenerative diseases, there is usually a subset of tissues or cell populations that are selectively susceptible to pathogenic agents [5,33,34,35].

Animal models (especially the widely used mouse models of NCL) and their characteristics allow researchers to develop disease phenotypes at the organismal level, as they largely reproduce certain disease-relevant phenotypes, with obvious species limitations [5,34,35,36,37,38,39,40,41,42,43,44]. With the advent of more accurate and precise technologies to generate mouse models with specific mutations, attempts have been made to replicate the most common human disease-causing mutations in mice, which would allow for a better representation and understanding of the pathological changes observed in human NCL [5,45,46,47,48]. Naturally, in addition to mouse models, larger animal models of NCL have been developed in dogs [5,49,50,51,52] and sheep [5,53]. These models have proved very useful for understanding the anatomical and pathophysiological spread of disease, as they more closely mimic the human disease and provide a means to test the delivery and dosing of therapeutic agents in a way that is not possible in mice [5].

The use of CRISPR-Cas9 has enabled the generation of a detailed and genetically comprehensive sheep model of CLN1 disease [5,54], as well as a porcine model of CLN3 disease [5,55]. In all likelihood, we can expect similar models to be developed for other forms of NCL [5].

### 2.2. Anatomical Regions Affected by NCL Pathology

A constant characteristic sign in NCL is cerebral and cerebellar atrophy co-occurring with enlargement of the lateral ventricles in the brain [5]. It should be noted, however, that atrophy is not a uniform process; thus, some regions are affected much earlier than others [5,23,56,57,58,59]. Studies in mouse models have shown that the thalamus and cerebellum are particularly vulnerable regions in various forms of NCL, and somatosensory regions of the cortex are affected earlier and more severely than motor regions [5,42,60,61,62,63]. Indicative changes include marked glial activation, accumulation of autofluorescent storage material (AFSM), and neuronal loss, as well as marked loss of interneurons in the cortex and hippocampus. The stage and progression of pathology has also been determined in larger animal models of NCL, confirming that these regions may, indeed, be clinically relevant [5,51,52,64,65,66]. Patients with NCL are often characterised by sensory and motor deficits [5,13]. Spinal cord involvement, as demonstrated in CLN1, may be responsible for this [67]. A study in animal models, as well as autopsy studies in humans, suggest that spinal cord pathology occurs with high frequency in many types of NCL [5,22,51,54,55,68].

Most patients with NCLs suffer from progressive loss of vision [3,5,13]. This has necessitated a detailed examination of the visual pathway, which has shown marked retinal degeneration in these patients [13,69,70]. In many forms of NCL, based on animal models, it is also often accompanied by optic nerve degeneration [5,71,72,73,74,75,76,77]. Furthermore, mouse models have been shown to exhibit significant pathology in central visual pathways within the dorsolateral geniculate nucleus of the thalamus and visual cortex in many forms of NCL [42,60,61,62], and it is likely that other retinal receptive nuclei are also affected. Another issue with mouse models is that rodent species rely much more on sensory information from the eyeballs than on visual information [78], which may explain why the somatosensory pathways through the posterior ventral thalamic nucleus to the barrel-field cortex are so severely impaired in the mouse models of NCL [61,62,63,79,80], even more so than the central visual pathways [42,60,61,62].

In NCLs, as in many other known storage diseases, findings provide evidence of significant cardiac pathology from both clinical observations and experimental studies [5,81,82,83]. These are best understood in CLN3 disease and also include evidence of autonomic nervous system dysfunction [84]. Furthermore, research reports also refer to the intestinal problems occurring in children with NCL [5,13].

Since most of the proteins in the NCL are widely expressed in different tissues and cell types, in all likelihood, we can suspect that the deficiency of these proteins also affects other organ systems in the body (including outside the CNS) [19,85,86]. Therefore, treating only the regions in the brain occupied by the disease process may not be sufficient to achieve therapeutic success. Making the identification of other, unexpected sites where pathology develops seem important. While the involvement of the brain by the disease process appears to be the primary concern, other disease effects, associated with other locations of disease development (although not to the same extent as the brain), will potentially also require therapeutic intervention. Treating these previously overlooked pathologies may provide additional clinical benefits, improving quality of life [5].

### 2.3. Cell Types in NCL Pathology

NCL affects different cell types [87,88,89,90]. Dysfunction and loss of the number of properly functioning neurons are characteristic features, bearing in mind that neuronal population types differ in their sensitivity to being affected [5,88]. One type of neuron that is more susceptible to damage in NCL is the interneuron population. The greatest loss of this population occurs in the thalamus, cerebral cortex, and hippocampus, where they account for the majority of neurons lost in the early stages of the disease [60,61,79].

Their greater vulnerability to damage may be due to their properties, mainly electrophysiological and bioenergetic [91]. What is clear in NCLs is that AFSMs are deposited in lysosomes, and their function is impaired; as it seems, the preserved normal lysosomal function is critical for the proper functioning of interneurons [47,92]. Interestingly, interneuron populations have also been shown to play an important role in the neurodegenerative pathways of various diseases, such as epilepsy [93], Alzheimer’s [94], amyotrophic lateral sclerosis (ALS) [95], and Parkinson’s [96].

Despite the fact that AFSMs accumulate in many cell types, NCLs have always been considered neuronal diseases, which is also reflected in their name, i.e., “neuronal ceroid lipofuscinoses” [5,83]. Nowadays, an early and significant effect of NCLs on other cells, which are also part of the CNS, is increasingly pointed out. These include astrocytes and microglia, which are observed in a state of activation, both biochemically and histologically [5,48,60,61,97]. Microglia activation generally occurs at a stage immediately preceding neuronal loss, and its location has a greater predictive value, in relation to the site of neuronal loss, than the site of storage material appearance [3,98]. Certainly, the question of whether glial activation contributes to neuronal population loss (which is a suggested pathophysiological pathway in other lysosomal or neurodegenerative disorders) still needs to be clarified [5,99,100,101]. Primary cell culture experiments investigating the properties of glial cells derived from mouse models of CLN1 and CLN3 have shown that astrocytes and microglia have significant functional and morphological defects, although they vary widely between these forms of NCL [5,25,26]. In co-culture systems, astrocytes and microglia have been shown to damage or negatively affect the survival of both healthy and mutant neurons [5,25]. These experiences highlight the role that glial cells appear to play in the pathogenesis of NCL. In addition to the innate immune changes evidenced by glial activation in the CNS, there is also evidence for a general humoral immune response in NCL [102,103], with a possible autoimmune component, particularly in CLN3 [5,104]. Authors should discuss the results and how they can be interpreted, from the perspective of previous studies and the working hypotheses. The findings and their implications should be discussed in the broadest context possible. Future research directions may also be highlighted.

### 2.4. Cellular Model

Mutations in the NCL involve the endo-lysosomal system. Lysosomes are responsible for recycling endogenous and internalized molecules, which are involved in maintaining tissue homeostasis [105,106]. They are also known to be involved in many cellular processes, such as nutrient sensing, calcium and bio-metal homeostasis, cell growth axonal transport, and synaptic homeostasis [105,106]. Because of this, autophagy function, which is essential for normal cellular homeostasis, is impaired in the NCL. Despite the disclosure of these disorders in various forms of NCL, the exact mechanism that leads to the consequent changes that cause cell death still remains unknown [47,80,107,108,109]. Similar disturbances in the autophagy mechanism have been revealed in other lysosomal disorders [110,111].

Animal models have also shown changes in synaptic vesicle density, exocytosis, and electrophysiological changes, which are indicative of synaptic dysfunction [62,65,108,112,113,114]. Despite the evidence that lysosomes are transported into the synapse to facilitate synaptic remodelling, pre-synaptic autophagy, synaptic vesicle sorting, and overall synaptic homeostasis [5,115,116,117], the exact mechanisms causing NCL to affect the lysosomal system, leading to synaptic dysfunction, are still not understood [5,115,116,117]. However, the question of synaptic dysfunction remains essential for a comprehensive understanding of the entire pathomechanism, mainly due to the fact that it often precedes the stage of neuronal loss. It is highly likely that this is due to altered conductivity within the neurons, as well as the presence of other signalling defects within cells [5,118]. Disease involvement of the (already mentioned) astrocytes and microglia is also one of the postulated mechanisms leading to loss of synaptic function [5,118,119,120,121].

The discovery of the cellular mechanisms responsible for controlling the expression of many endo-lysosomal proteins is undoubtedly one of the major achievements of molecular biology. These include the discovery of the coordinated lysosomal expression and regulation (CLEAR) transcription initiation site, the EB transcription factor (TFEB), and its phosphorylation by mTORC1. Of particular relevance to the pathophysiology of NCLs was the demonstration of dysregulation of this pathway in many NCL types [5,122,123,124,125,126]. Preclinical studies in a mouse model provided evidence that the aforementioned dysregulation may be a viable therapeutic target [127,128], mainly because an upregulation of TFEB increases the overall cellular clearance, which may bypass the existing lysosomal dysfunction [127,128].

### 2.5. Genotype–Phenotype Correlations

All of the groups of neuronal ceroid lipofuscinoses are monogenic disorders, so each represents a distinct disease entity. Genes whose mutations are responsible for the occurrence of NCL encode seemingly unrelated proteins, including the soluble lysosomal enzymes and membrane proteins located in various organelles, including the lysosome [3,19,129,130]. All types of NCL are inherited autosomal recessively, except neuronal ceroid lipofuscinosis type 4 (CLN4) [3,129]. For most NCLs, there is a recognizable classical disease phenotype associated with the complete loss of gene function, due to intracellular mislocalization or degradation of the mutant protein [129]. In addition, there are forms of the disease that may have a more chronic course, and some of the predicted classical phenotypes may not be present, as a result of milder mutations that do not cause complete arrest of protein function [3,24]. There are examples of mutations associated with a specific phenotype, such as a missense mutation in CLN8 or a 1 kb intragenic deletion that underlies the most common form of NCL, i.e., juvenile CLN3 disease [3,24,129]. The most common mutations are a 1 kb deletion in CLN3 and two mutations in CLN2 [3,23,24].

## 3. Clinical Features

The clinical picture in which NCL was diagnosed in the past was the result of careful analysis of a compilation of case reports; nowadays, natural history reports, regarding the course of the disease in patients, continue to be used to advance knowledge of NCL and to prepare clinical trials [28,131,132,133,134,135]. In the clinical setting, the age and onset of symptoms continue to play a key role in suspecting NCL and, ultimately, pursuing confirmatory genetic testing for the disease. All patients with NCL, except those with the rare congenital form (neuronal ceroid lipofuscinosis type 10 (CLN10)), have normal psychomotor development before the onset of first symptoms [3,13]. In most patients, the time of onset of the first noticeable symptoms of the disease is during childhood; however, in some, it can be as long as 60 years or more. NCL is a collection of disparate subtypes, but typical clinical manifestations of NCL include progressive loss of vision, dementia, psychomotor stunting, inability to reach normal developmental milestones and/or developmental regression, behavioral problems, progressive brain atrophy, seizures, cognitive decline, and motor skills [3,16,19,24,130,136,137,138].

Cognitive decline in young children occurs rapidly, while adolescents and adults show a much slower rate, allowing for normal life and education. Cognitive decline occurs as a two-phase problem [139]. Early in the disease, children’s developmental trajectory slows; they acquire new learning more slowly than their peers but often remain within the normal range for some time [139]. They go on to plateau and then begin to lose the cognitive competences they have acquired during the earliest months and/or years of their lives [139]. This is often the time at which the diagnostic investigations are triggered. In CLN2 disease, however, many children show a delayed acquisition of cognitive skills at a very early stage; their profiles are characteristically uneven, with delays being more evident in the domain of expressive language, compared with motor skills [139]. In CLN3, the rate of disease progression is commonly slow, and children can attend school, with support for the intellectual, visual, and, sometimes, behavioral difficulties. There is no evidence, to date, of an uneven developmental profile early in life and before the onset of progressive vision loss [139].

Symptoms of the disease can also affect other regions, often not directly related to the central nervous system. This is the case with CLN3, where cardiac involvement is often described in adolescent and adult patients [84,140]. This problem is amenable to treatment by implanting a pacemaker, when the patient’s psychomotor performance has improved [3,139].

Among the first symptoms in the classic (age of onset 6–24 months) and late (age of onset 2–5 years) infantile phenotypes is a slowing of psychomotor development, quickly followed by developmental arrest, gradual loss of acquired psychomotor skills, onset of epilepsy, and, finally, loss of vision [13]. Unfortunately, these disorders, particularly the regression of psychomotor abilities, are often misdiagnosed as side effects of the drugs used (for example, antiepileptic drugs), which delays the diagnosis of NCL and, by further exacerbating developmental defects, worsens the child’s condition. On the other hand, in the juvenile phenotype (age of onset 5–7 years), the first symptoms are usually the progressive loss of vision, followed by the development of dementia and behavioral changes, and, finally, the loss of motor skills and epilepsy in early adolescence. In the recessive adult phenotype (over 16 years of age), also referred to as Kufs disease, visual loss is usually absent, and patients develop progressive myoclonic epilepsy (type A) or dementia with motor impairment (type B), which usually begins between 16 and 50 years of age, but most commonly around age 30 [3,24].

The delayed development of expressive language, also known as expressive language disorder, is revealed as the first symptom indicating the deterioration of psychomotor function in 83% of patients with classic late-onset CLN2 disease—this may be an indication to screen the child for CLN2 and allow for early diagnosis [3,134,141].

One of the persistent symptoms of NCL also includes epilepsy. This is very troublesome, as it is characterized by treatment resistance in almost all patients. The use of more than two antiepileptic drugs is ineffective, as it only exacerbates their side effects, without controlling or reducing the number of seizures [3,13]. In the pharmacotherapy of epilepsy occurring in the CLN2 and CLN3 subtypes, lamotrigine and valproic acid are the recommended drugs [3,142,143], whereas drugs such as phenytoin, gabapentin, vigabatrin, or carbamazepine may cause an increase in myoclonic seizures in these patients [3,142,143]. The frequency and severity of epileptic seizures is particularly high in late infantile CLN2, which continues until the late stages of the disease [3,141]. The situation is different, for example, in infantile neuronal ceroid lipofuscinosis type 1 (CLN1), where seizure frequency tends to decrease in the later stages of the disease, or in patients with classic juvenile CLN3, where seizures are rare and only moderate severity occurs in the later stages of the disease [3,141,142,143]. As the disease progresses, the benefits and harms of specific medications should be reconsidered, as these drugs, while tolerable and effective in the earlier stages of the disease, may prove ineffective after progression and cause only side effects [143].

Typical neurological manifestations of NCL also include motor symptoms, such as ataxia, dysphagia, myoclonus, chorea, tremors, and dystonia [3]. These especially occur in the classic and late infantile phenotypes [3,13]. In addition, parkinsonism is also present, especially in juvenile CLN3 disease, and some stereotypic movements have also been described in various types of NCL with late infantile and juvenile onset [3,13]. Table 3 shows the clinical characteristics of the different subtypes of neuronal ceroid lipofuscinoses.

## 4. Diagnosis

Many known childhood neurodegenerative diseases have similar symptoms, so the delayed diagnosis of NCL is unfortunately common [16,144]. Definitive diagnosis can be problematic in infants and young children, in whom a thorough neurological and ophthalmic evaluation requires a skilled and knowledgeable specialist. NCL occurs after a period of apparently normal development, despite the absence of a functional protein that is important for brain function; thus, it must be concluded that there is a small therapeutic window in which effective interventions can halt and/or prevent disease progression. Consequently, early diagnosis is crucial for optimal therapeutic outcomes, and prospective studies must also include those patients with only modest disease progression. Consequently, knowledge of the clinical features, which unfortunately are highly nonspecific, seems to be a necessity for making a suspected diagnosis and carrying out the diagnostic process, in order to be able to definitively confirm the diagnosis as soon as possible and to incorporate the treatment adapted to it.

However, there is no single, universally valid diagnostic algorithm for neuronal ceroid lipofuscinoses. The gold standard diagnostic tool for NCL is genetic testing, in order to detect and confirm the presence of the mutated gene; however, these tests are expensive and may take months to analyze and provide results. It is important to keep in mind, however, that this is not one of the first tests of choice, neuronal ceroid lipofuscinosis is a very rare disorder; before the clinician can guide his diagnostic process toward NCL, it is necessary to first rule out other, much more common potential causes of the condition that the patient is experiencing. Tests that are used to diagnose NCL in a patient with suspected clinical symptoms include enzyme tests, neuroimaging studies (e.g., magnetic resonance imaging or computed tomography), visual evoked potentials, electroencephalography (EEG), and other electrophysiological studies [16,145,146,147]. Genome analysis will certainly identify the known pathogenic variants; however, in some cases, detection of the common disease-causing mutations is not confirmed. In this case, we expand the diagnosis with biochemical assays. When biochemical impairment is found, the diagnostic search for changes in cryptic gene regions (introns, untranslated regions, etc.) is extended [139].

### 4.1. Genetic Testing

Technological advances and new DNA testing technologies, mainly concerning genomic sequencing, now make it possible to study multiple genes in a single step, regardless of presentation [3]. Whole exome, whole genome, or direct Sanger sequencing are the most reliable analytical tools for the accurate identification of mutations in patients [16,129,147,148] and should be performed in such rare diseases because overlap with other neurodegenerative disorders is blurred. These methods are particularly helpful for patients with clinical variants and/or unusual or novel mutations. The significant heterogeneity of this group of patients, due to the vast differences in age of onset, clinical presentation, severity, and disease course, makes genetic testing the only universal tool at our disposal, with nearly 100% sensitivity, that clinicians and researchers can use to identify the specific genetic mutations that may cause protein dysfunction and confirm the diagnosis of neuronal ceroid lipofuscinoses [16,129,147,148].

Currently, in the diagnostic process, genes whose mutations are responsible for the occurrence of NCL are part of the panels designed to examine the genes underlying a larger group of syndromically and nonsyndromically inherited epilepsies. Some common mutations can be tested using DNA-based assays. This can expedite the earlier diagnosis of NCL before the onset of other symptoms, as well as provide a genetic diagnosis for milder clinical phenotypes or variants. Because DNA sequencing leads to the description of many genetic variants, the genetic cause of atypical disease will become clearer in some cases. Some patients who were previously diagnosed with NCL may turn out to have atypical forms of other diseases and vice versa. Detection of the carrier is not possible by histology, and it is unreliable by enzymatic analysis; mutation analysis should always be relied upon.

There is a mutation database that catalogs all known variant mutations of the NCL genes, which includes nearly 446 disease-causing mutations, and it should be noted that new disease-causing gene mutations are still being discovered [16,20,129].

### 4.2. Enzyme and Microscopic Assays

Biochemical assays for the activity of four lysosomal enzymes that are mutated in various forms of the disease (PPT1, TPP1, CTSF, and cathepsin D (encoded by *CTSD*)) can rapidly confirm their deficiencies and are among the conclusive methods for diagnosing CLN1, CLN2, and CLN10, respectively [16,20,139,149,150,151,152,153]. These tests can be performed using saliva obtained from the patient, a blood sample, or a dried blood spot [3,20]. Enzyme tests should always be used in cases with an unusual appearance or later onset, and all diagnoses should be supported by DNA sequencing and mutation analysis, if possible [20,130]. For PPT1 testing, patient samples, including fibroblasts, leukocytes, amniocytes, dried blood spots, and chorionic villi, are mixed with a synthetic substrate that is hydrolyzed by the active PPT1 enzyme to release a detectable fluorophore [16,20,152,153]. The absence of a fluorophore confirms CLN1 disease. Similarly, TPP1 and CTSD activity assays use fluorogenic synthetic substrates conjugated to Ala-Ala-Phe and conjugated to hemoglobin, respectively, in which the active enzyme cleaves the substrate to activate the fluorophore [16,20,154,155,156]. Each of these tests can be considered rapid and reliable tools for assisting clinicians in the initial diagnosis of the disorder, as well as in distinguishing its potential subtypes [16,20]. In adults with nonspecific mental, motor, or behavioral disorders, in whom NCL is suspected, the first line of testing includes the just-mentioned enzymatic tests for PPT1, TTP1, CTSD, and CTSF, which, when normal, should prompt the clinician to perform ultrastructural testing [2,16,20]. If storage material is present, genetic testing should be initiated for autosomal recessive (CLN6, CLN11/GRN, and CLN13/CTSF) and dominant (CLN4/DNAJC5) NCL; if negative, all other NCL genes should be tested [2,16,20]. The occurrence of disease in a newborn with severe epilepsy and microcephaly should suggest CLN10 disease as a possible diagnosis. Enzymatic testing for CTSD (CLN10) should be the first step. If negative, further or concurrent enzyme testing for PPT1 and TTP1 should be performed before more invasive biochemical testing.

In young children (>6 months) with unexplained epilepsy and developmental arrest, CLN1 and CLN2 are most likely to be affected. If enzyme tests for PPT1 and TTP1 are negative and electron microscopy shows typical storage material, genetic testing for CLN5, CLN6, CLN7/MFSD8, CLN8, and CLN14/KCTD7 should be considered [2].

In countries where genetic and biochemical testing are not readily available, skin biopsy samples can also be obtained, with the lipopigment accumulation assessed, which is a pathological feature of these disorders [16,23,157]. Microscopic analysis of ultrastructural patterns of cellular deposits helps divide patients into the possible subtypes of NCL. Lipopigment morphotypes usually correlate strongly with the genotype [16,35,158]; however, the genotype does not always coincide with the clinical presentation of the disease. For this reason, storage deposits can be used as a confirmation of neuronal ceroid lipofuscinosis subtypes, rather than as a diagnostic tool, with different morphotypes indicating different forms of the disease [2,16,23]. Ultrastructural examination of a skin biopsy or blood sample can be helpful in confirming the NCL disease subtype for atypical forms that are not enzyme deficient or genetically undiagnosed. Extracerebral storage is readily detectable in childhood NCL, but not necessarily in NCL occurring in adulthood [21,24].

In addition, in patients suspected of having classic juvenile CLN3 disease, such as a school-aged child with rapid visual loss between 4 and 7 years of age, one should look for vacuolated lymphocytes. This feature, characteristic of CLN3, can be assessed by a simple blood smear, and it can be helpful in the differential diagnosis of patients [2,16,19,138]. In contrast, if no lymphocyte vacuolization is found and the PPT1, TPP1, and CTSD tests are negative, it is advisable to perform the aforementioned skin biopsy to assess whether typical storage material for NCL is present. If so, genetic testing for CLN5, CLN6, CLN7/MFSD8, CLN8, and CLN12/ATP13A2 is indicated [2].

### 4.3. Neuroimaging

Brain MRI findings may appear normal in the early stages of disease or show some nonspecific signs, such as periventricular intensity changes in early CLN2 disease [3,159]. Although MRI is not sensitive or specific for early diagnosis, it is an excellent tool for objectively monitoring the progression of brain changes, especially with changes in resolution and processing, and neuroimaging techniques, such as diffusion tensor imaging, allow for the assessment of white matter pathway disorganization and atrophy [3,23,54,55,56,57,58,59]. Magnetic resonance imaging in advanced stages of the disease can mainly visualize cortical gray matter atrophy, cerebellar atrophy, and secondary ventricular enlargement [3,23,54,55,56,57,58,59]. The results in patients with CLN2 disease have shown that the loss of cortical gray matter volume with age can be a sensitive biomarker for monitoring disease progression [2,58,159].

### 4.4. EEG Monitoring and Other Electrophisiological Studies

EEG can be helpful in the early diagnosis of some NCLs. For example, characteristic posterior spike waves after low-frequency photostimulation have been reported in more than 90% of patients with early-stage CLN2 disease [3,160]. The finding of posterior spike waves after photostimulation in a child under 5 years of age with new onset seizures should be a reason to initiate testing for CLN2 disease [3,160,161]. Photosensitivity with low-frequency stimulation has also been described in patients with CLN6 (neuronal ceroid lipofuscinosis type 6) disease, especially in adults with CLN6 (type A) disease. [3,137,162,163]. In forms of NCL that progress rapidly, such as infantile CLN1 disease, early abnormalities disappear as neurons die, leading to a characteristic flat EEG in advanced disease [3,13]. Importantly, in NCL, due to the underlying neurometabolic disease, taking antiepileptic drugs does not lead to seizure control and the normalization of EEG results [3,13], possibly because the cells have died (or are dying), which itself leads to seizures. EEG monitoring may be useful in detecting signs of encephalitis, which may be a rare consequence of antiepileptic drugs, such as valproate, in advanced, late-infantile CLN2 disease [3,143] or a theoretical allergic reaction to new treatments [3].

Another of the electrophysiological studies that are crucial in rare diseases, such as NCL, is photoparoxysmal response (PPR). It was first described in the 1980s [164,165], and subsequent studies have confirmed this neurophysiological finding [165,166,167,168,169]. The PPR seen in the NCLs is characterized by an occipital spike-and-waves response to the photic stimuli [165]. However, it should also be highlighted that patients do not have the same susceptibility to PPR in all NCL variants—it has been reported more frequently in CLN2, CLN5, and CLN6 diseases [165]. In CLN2 disease, PPR has been reported from 27 to 93% of patients [165,166,168,169,170]. In a series of 14 CLN2 cases, serial EEG showed PPR in 93% of patients [165,166]. PPR was evident from the first EEG in 43% of patients; it was documented at low (1–3 Hz) stimulation frequencies in 69% of patients and took the form of a flash-per-flash response in 69% of patients [165,166]. Interestingly, in advanced stages of the disease, PPR was associated with massive myoclonic jerks [165,166]. In approximately half of patients (54%), PPR disappears over time [164,165]. Changes in PPR over time reflect changes in the grey matter caused by the progression of neurodegenerative disease [165,166]. It is also worth mentioning that PPR is one of the earliest pieces of evidence of a neurodegenerative disorder, even before the onset of cognitive and motor regression [165,171].

To date, little is known about changes in visual evoked potentials (VEPs) during the course of disease in NCLs [165]. It is known that so-called giant VEPs appear as the disease progresses. They are abnormally broad and of high amplitude; their presence is a marker of cortical hyperactivity, similar to PPR [165,167]. Abnormal VEPs were found in approximately 75% of CLN2 patients [165,170]. The majority of patients (89%) had an early reversal of the high-amplitude pattern in the VEP, while a small number of patients showed a bifid waveform, which is associated with central scotoma, indicative of maculopathy or macular pathway dysfunction, such as optic nerve atrophy [165,172]. As the disease progresses further, the amplitude of the VEP decreases, as it does in other neurodegenerative disorders [165].

In addition to visual evoked potentials, there are also somatosensory evoked potentials (SEP), which are less well-studied in NCL than VEPs. There are sporadic cases across different forms of NCLs [149,165,167,173,174], where it is highlighted that the presence of high-amplitude evoked potentially giant SEPs, which are the expressions of cortical hyperexcitability, due to neuronal degeneration [165,167].

Progressive loss of vision is one of the classical symptoms of NCL. It affects all forms; in the large majority of cases, it occurs as one of the early clinical signs [139,165]. Retinal structures, visual pathways, and visual cortices are affected [139]. Both ganglionic neurons and receptor cells (cones and rods) are involved [139]. For this reason, an electroretinogram (ERG) is also one of the electrophysiological studies [139,165]. It is a tool that allows for the monitoring of retinal involvement, and its use gradually disappears as the disease progresses, depending on the variants of NCL [165,175]. The use of ERG might be implemented in the future to evaluate the efficacy of experimental treatments with intravitreal therapies [165].

### 4.5. Prenatal Diagnosis

Prenatal diagnosis may be offered to families with prior NCL disease. Preimplantation genetic diagnosis [21,174] or a combination of enzyme assay and mutation analysis, perhaps with ultrastructural examination of chorionic samples obtained at 12–15 weeks gestation, may provide a rapid diagnosis [3,20].

However, genetic testing and enzyme activity assays are now the standard. These methods are readily available in most developed countries and can reliably be performed prenatally using amniotic fluid or fetal cells [16,23,149,175,176,177].

In Table 4, we have presented the subtypes of neuronal ceroid lipofuscinosis and their possible deviations in the results of tests that are performed during the diagnostic process (neuroimaging, EEG monitoring, and visual and microscopic examinations).

## 5. Conclusions

NCLs include a group of rare, fatal, neurodegenerative, lysosomal storage disorders. They predominantly affect the pediatric population, but there are also cases with disease onset in adulthood. Specific mutations cause the onset of the disease and often correlate with the clinical phenotype. Some variability in the clinical phenotype can, therefore, be explained by different levels of residual protein function. However, variability between families, and even between siblings, shows that co-inheritance of other genetic variations may affect the disease phenotype. The typical clinical manifestations include progressive loss of vision, mental and motor deterioration, epileptic seizures, behavioural problems, cognitive decline, and dementia. All of these significantly reduce the patients’ quality of life. Through functional impairment and the resulting dependence on caregivers and facilities, the exclusion of patients from society continues to progress. Understanding the molecular basis of NCL seems to play a key role, as knowing the exact cause and pathomechanisms can lead to effective causal treatment. This is extremely important, as current treatment options are only symptomatic and focus on delaying disease progression. It is worth noting the international cooperation in creating a database of patients and mutations, which is of great importance for accessing information on each type of NCL, as well as for planning future clinical trials.

## Figures and Tables

**Table 1:** Types of NCL.

A	Congenital NCL
	Babies are born with microcephaly as the disease begins in utero [21,27].
B	Infantile NCL
	Seizures and the loss of motor function appear between the ages of 6 and 18 months, with the loss of psychomotor skills, including speech. Signs of regression, accompanied by the onset of epilepsy and a progressive loss of vision. Hyperexcitability. Restlessness and poor sleep. After the age of 15 to 20 months, the acceleration of symptoms occurs, leading to microcephaly, truncal ataxia, dystonic features, choreoathetosis, and myoclonic jerks. By the age of 24 months, children become blind and lose all cognitive and active motor skill. Death usually between the ages of 9 and 13 [21,28].
C	Late infantile NCL
	Developmental delay, ataxia, and seizures appear between the ages of 2 and 4 years old and progress rapidly to loss of motor, cognitive, and language functions, ultimately becoming behaviorally abnormal and demented [21,28].
D	Juvenile NCL (JNCL)
	The most common type of NCL. Symptoms occurring between 5 and 10 years old—commonly associated with the progressive loss of vision and seizures. Learning difficulties and motor disturbances, including extrapyramidal and less prominent pyramidal involvement (rigidity, bradykinesia, slow steps with flexion in hips and knees, and shuffling gait), appear around puberty. Death during their third decade [21,28].
E	Adult NCL
	Symptoms are less severe and progress more slowly. The clinical picture is characterized by generalized tonic seizures, myoclonus, and prominent dementia. Associated features include speech problems, cerebellar dysfunction, and parkinsonism [21,29,30].

**Table 2:** NCL diseases and encoded genes [19,21,31,32].

Disease	Gene/Protein	Age of Onset	Protein Function
CLN1	*PPT1* (palmitoyl protein thioesterase 1)	6–18 months	Palmitoyl-protein thioesterase activity plays a critical role in the degradation of lipid-modified proteins via removing fatty acid residues from cysteine residues.
CLN2	*TPP1* (tripeptidyl peptidase 1)	2–4 years	Serine protease activity prevents intralysosomal accumulation of storage material and neuronal loss.
CLN3	*CLN3*, lysosomal/endosomal transmembrane protein	4–10 years	Predicted function as a pH regulator and modulator of vesicular trafficking and fusion that promotes cellular homeostasis and neuronal survival.
CLN4	DNAJC5/CSPα (cysteine string protein α)	>18 years	Involvement in exocytosis and endocytosis functions plays a regulatory role in ATPase activity and assists in folding proteins in synaptic vesicles.
CLN5	Soluble lysosomal protein	4–7 years	Glycoside hydrolase activity modulates vesicular trafficking.
CLN6	Transmembrane protein of endoplasmic reticulum	18 months–6 years	Precise function remains unclear but is linked with intracellular trafficking and lysosomal function.
CLN7	MFSD8 (major facilitator superfamily domain-containing 8), lysosomal transmembrane protein	2–6 years	Predicted transmembrane transporter function plays a role in preventing neuronal loss, robust accumulation of lipofuscin, reactive gliosis, and degeneration and storage accumulation in the retina.
CLN8	Transmembrane protein of endoplasmic reticulum	2–7 years (Turkish variant late-infantile NCL)	Aids in lysosomal biogenesis, through transportation from the ER to the Golgi complex, as well as in the regulation of lipid metabolism.
		5–10 years (northern epilepsy)	
CLN10	CTSD (cathepsin D)	In utero	Aspartic protease functions in an unknown neuroprotective mechanism.
CLN11	PRGN (progranulin)	20–25 years	Known roles in inflammation, embryogenesis, cell motility, and tumorigenesis.
CLN12	ATP13A2	13–16 years	Regulation of ion homeostasis.
CLN13	CTSF (cathepsin F)	>18 years	Loss of lysosomal cysteine protease activity leads to the deterioration of motor function and reduced brain function.
CLN14	KCTD7 (potassium channel tetramerization domain-containing protein 7)	8–24 months	Modulation of potassium ion channel activity.

**Table 3:** Major clinical manifestations [3,4,13].

Disease	Clinical Manifestation
CLN1	Cognitive and motor decline Ataxia Choreoathetosis Myoclonus, epilepsy Progressive loss of vision Decelerated head growth Irritability Hyperexcitability Death by 10 years
CLN2	Cognitive decline Motor coordination loss Ataxia Choreoathetosis Myoclonus, spasticity Dystonic movements Epilepsy Progressive loss of vision Behavioural disturbances Death in early adolescence
CLN3	Progressive loss of vision Pigmentary retinopathy Motor e cognitive decline Epilepsy Behavioural disturbances Spasticity, myoclonus Death in the second decade
CLN4	Cognitive and motor decline Epilepsy, myoclonus Ataxia, tremors Behavioural disturbances No visual impairment Death within 15 years after onset
CLN5	Psychomotor regression Ataxia Motor coordination loss Epilepsy Progressive loss of vision Macular degeneration Death between 13–30 years
CLN6	Motor skills loss Dysarthria Ataxia Epilepsy Progressive loss of vision Death in the second decade
CLN7	Cognitive and motor decline Ataxia, myoclonus Epilepsy Progressive loss of vision Behavioural disturbances Death in the second decade
CLN8	Cognitive and motor decline Ataxia Myoclonus Epilepsy Progressive loss of vision Retinopathy Behavioural disturbances Death within the sixth decade
CLN10	Microencephaly Hypotonia Absence of reflexes Respiratory impairment at birth Epilepsy Death within hours after birth
CLN11	Rapid progressive retinal dystrophy and progressive loss of vision Cognitive decline Epilepsy Myoclonus Ataxia
CLN12	Learning disorders Loss of speech Loss of coordination Ataxia, myoclonus Epilepsy Spasticity Behavioural disturbances Muscular atrophy No visual impairment
CLN13	Memory deficits Behavioural disturbances Tremors, bradykinesia Ataxia, rigidity Dysarthria Epilepsy No visual impairment
CLN14	Motor decline Epilepsy Myoclonus Ataxia Dysarthria Optic atrophy Progressive loss of vision Behavioural disturbances

**Table 4:** NCL subtypes and abnormalities in diagnostic test results [3,4,16,20].

Disease	Neuroradiological Imaging Findings	EEG Abnormalities	Visual Changes	Microscopic Findings
CLN1	Hyperintense, periventricular high-signal rims of white matter; decreased NAA and increased choline; and severely enlarged lateral ventricles	Loss of sleep spindles at ~2 years; attenuated reaction to passive eye opening and/or closing; background activity disturbances; and reduced amplitude	Optic atrophy; unrecordable ERG at 4 years; and blindness	GRODs
CLN2	Infratentorial atrophy; hypointense thalamic nuclei; decreased NAA; increased myoinositol and Glu:Gln ratio in the white matter; and severely enlarged lateral ventricles	Occipital spike in response to slow flash; irregular slow activity; focal spikes; and absence of sleep spindles	Progressive loss of vision and diminished ERG	CLPs
CLN3	Cerebellar atrophy; enlarged third ventricle and cerebral sulci; and hypointense thalamic nuclei	Progressive background disorganization; and spike-and-slow-wave complexes	Progressive loss of vision and pigmentary retinopathy	FPPs and vacuolated lymphocytes
CLN4	Parieto-occipital cortical atrophy; cerebellar atrophy; hyperintense periventricular areas; and corpus callosum thinning	Slow background; polyphasic spikes; and slow-wave discharges	No visual impairment	GRODs; CLPs; and FPPs
CLN5	Cerebellar atrophy; diminished signal intensity in thalamic nuclei; and increased signal intensity in periventricular white matter and internal capsule	Occipital spikes in response to slow flash	Progressive loss of vision and macular degeneration	GRODs; CLPs; and FPPs
CLN6	Deep cortical layer-specific neuron loss and cerebellar atrophy	Background slowing; and high-amplitude discharges, in response to photic stimulation	Progressive loss of vision	CLPs; RLC; and FPPs
CLN7	Cerebellar atrophy; corpus callosum thinning; and hypointense thalamic nuclei	Occipital spikes; and background slowing	Progressive loss of vision	CLPs; RLC; and FPPs
CLN8	Cerebellar atrophy; corpus callosum thinning; and hyperintensity of white matter	Slow background; components of high amplitude; and epileptiform discharges	Retinopathy; visual decline at around 4–6 years of age; and ERG absent	GRODs; CLPs; and FPPs
CLN10	Diminished head growth in utero; myoclonic fetal seizures; enlarged lateral ventricles; hypointense cerebral and cerebellar white matter; and decreased NAA and increase in myo-inositol	Completely depleted EEG pattern	ND	GRODs
CLN11	Cerebellar atrophy	Polyspike-wave discharges with posterior emphasis; and severe attenuation of rod and cone responses	Progressive loss of vision and retinal dystrophy	FPPs
CLN12	Cortical and subcortical atrophy; decreased glucose use in grey matter, especially the thalamus and posterior association cortex	ND	No visual changes	GRODs
CLN13	Cerebellar atrophy; frontal and parietal cortical atrophy; and periventricular hyperintensities	No epileptiform activity	ND	FPPs
CLN14	Cortical and cerebellar atrophy; and corpus callosum thinning	Slow dysrhythmia; multifocal high-amplitude epileptiform discharges; photosensitivity; and occipital spikes	Progressive loss of vision; diminished pupillary light reflex; and optic atrophy	GRODs; CLPs; RLC; and FPPs

Abbreviations: NAA = *N*-acetyl aspartate; ERG = electroretinogram; GRODs = granular deposits; CLPs = urvilinear profiles; FPPs = fingerprint profiles; RLC = rectilinear complex; ND = not determined.

## Data Availability

Not applicable.
